# Trait‐based diatom functional diversity as an appropriate tool for understanding the effects of environmental changes in soda pans

**DOI:** 10.1002/ece3.5897

**Published:** 2019-12-10

**Authors:** Csilla Stenger‐Kovács, Edina Lengyel, Krisztina Buczkó, Judit Padisák, János Korponai

**Affiliations:** ^1^ Department of Limnology University of Pannonia Veszprém Hungary; ^2^ Department of Botany Hungarian Natural History Museum Budapest Hungary; ^3^ MTA Centre for Ecological Research Danube Research Institute Budapest Hungary; ^4^ Department of Water Supply and Sewerage Faculty of Water Science National University of Public Service Baja Hungary; ^5^ Department of Environmental Sciences Sapientia Hungarian University of Transylvania Cluj‐Napoca Romania

**Keywords:** conservation, diversity indices, ecological status, environmental constrains, saline ecosystems

## Abstract

Saline lakes, among the most seriously endangered ecosystems, are threatened due to climate change and human activities. One valuable feature of these environments is that they constitute areas of high biodiversity. Ecologists are, therefore, under great pressure to improve their understanding of the effects of natural and anthropogenic disturbances on the biodiversity of saline lakes. In this study, a total of 257 samples from 32 soda pans in Central Europe between 2006 and 2015 were examined. The effects of environmental variables and of geographical and limnoecological factors on functional diversity were analyzed. Furthermore, the explanatory power of the trait‐based approach was assessed, and the applicability of the indices for biomonitoring purposes was determined. It was found that low habitat heterogeneity and harsh environments lead to the selection of a small number of suitable traits, and consequently, to a naturally low level of functional diversity. Anthropogenic activities enhance diversity at functional level due to the shift toward freshwater characteristics. On the regional scale, the effects of the region and status (natural, degraded, reconstructed) on diatom functional diversity were significant and more pronounced than that of the environmental and other limnoecological factors. The degree of variance found in functional diversity ascribed to environmental variables is five times greater in the case of the application of a trait‐based approach, than when a taxonomic one is employed in the literature. Each of the tested functional diversity indices was sensitive to the most important environmental variables. Furthermore, these were type‐specific and proved to be more complex indicators than taxonomic metrics. It is possible to suggest four functional diversity indices (FGR, FRic, FDis, and FDiv) which emphasize their independence from substrate and seasonal variations for ecological status assessment and conservation planning.

## Graphical abstract

In pristine soda pans, low habitat heterogeneity and harsh environment led to the selection of small number of suitable traits. Variance of the functional diversity indices explained by the environmental variables was five times higher applying trait‐based approach than taxonomic one. Functional diversity indices were type specific and more complex indicators than taxonomic metrics.

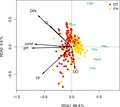

## 1 INTRODUCTION

In recent decades, biodiversity research has focused mostly on species richness and diversity metrics based on taxa as taxonomic units (e.g., Robinson, Rushforth, & Minshall, 1994; Tews et al., 2004). These diversity metrics have been applied as common indicators of environmental impacts (He, Jiang, Tang, & Cai, 2015), in which the species correctly identified under the microscope have served as a basis for the analyses (Korponai et al., 2019). Nowadays, a new generation method, DNA metabarcoding, has established the conditions for the identification of operational taxonomic units (OTU) in many hundreds of samples simultaneously (Taberlet, Bonin, Zinger, & Coissac, 2018). This method seems likely to broaden our knowledge of biodiversity and with phylogenetic estimation of OTU ecological profiles it will move closer to functional biomonitoring (Keck, Vasselon, Rimet, Bouchez, & Kahlert, 2018).

Recently, trait‐based approaches using functional trait units have drawn attention to the ecological and biological importance of the species (Schneider et al., 2017). In this sense, improved or more accurate predictions of ecosystem functions may be expected than were available using the taxonomic approach (Thompson, Davies, & Gonzalez, 2015). It was for this reason that the usefulness of this approach has been rapidly recognized and applied by ecologists. This recognition initiated an intensive search to discover the nature of the relationship between traits and habitat properties (Schneider et al., 2017) via the identification of the drivers of the diversity patterns. However, functional diversity metrics (He et al., 2015) have rarely been used recently, even though they promise to improve our knowledge of community and ecosystem responses to environmental changes at different scales (Péru & Dolédec, 2010). Furthermore, functional diversity can be a good indicator of ecosystem stability (Schneider et al., 2017) and can be strongly correlated with DNA‐based phylogenetic diversity (Li et al., 2019) through ecological traits as phylogenetic signals (Keck, Rimet, Franc, & Bouchez, 2016; Keck et al., 2018; Winter, Devictor, & Schweiger, 2013). Consequently, functional diversity can play an effective role in conservation management using phylogenetic tools (Webb, Ackerly, McPeek, & Donoghue, 2002).

Functional approaches require simpler data than do traditional taxonomic approaches, and at first glance, this may appear to reduce ecological information. Nonetheless, this approach is capable of increasing the variance which can be explained in a community by the environmental variables (Abonyi et al., 2018). This is because of their sensitivity and consistent response to distinct ecological drivers (Tolonen, Leinonen, Marttila, Erkinaro, & Heino, 2017). Moreover, complementary functional diversity indices are available, which are capable of indicating different aspects of ecosystem functioning and environmental changes (e.g., Mouchet, Villéger, Mason, & Mouillot, 2010; Schmera, Erős, & Podani, 2009).

In aquatic ecosystems, trait‐based methods have received intense attention in recent years (Endrédi, Jordán, & Abonyi, 2018; Wu et al., 2017) since they can be used independently of ecoregions (Dolédec & Statzner, 2008) and provide deeper insights into the functional and structural characteristics of communities (Verberk, Noordwijk, & Hildrew, 2013) through different environmental filters. Trait‐based approaches can provide an easier, faster, and more general understanding (Flynn, Mirotchnick, Jain, Palmer, & Naeem, 2011) of community organization than traditional taxonomical methods.

The application of functional traits and diversity indices as indicators of stressors of aquatic organisms is scarce (Ding et al., 2017). Only a few studies connecting structural patterns to the primary production are to be found (Niyogi, Lewis, & McKnight, 2002; Rowe, Sánchez‐España, Hallberg, & Johnson, 2007), and especially in the case of phytoplankton (Abonyi et al., 2018; Török et al., 2016) and benthic algal communities (B.‐Béres et al., 2019; Cibils, Principe, Márquez, Gari, & Albariño, 2015). However, diatoms are one of the most understudied groups of biota from this point of view (Alahuta et al., 2018), despite the possibility that diatom trait diversity (e.g., thickness of the valves, size, morphology or life strategies, and linking ability) may have a crucial role in environmental processes such as the ocean carbon pump (Tréguer et al., 2018).

Saline lakes are among the most vulnerable types of aquatic ecosystems due to the environmental threat generated by diverse human impacts (e.g., drainage and immoderate pumping of ground water) and climate change (Williams, 2002). The maintenance of the natural hydrological cycles and natural characteristics of these endorheic shallow lakes is key ecological and conservation tasks (Stenger‐Kovács et al., 2014). In contrast to typical saline waters, which are often permanent and characterized mainly by chloride ions, astatic soda pans are mostly dominated by bicarbonate (Boros & Kolpakova, 2018) and are to be found across Africa, Europe, Asia, Australia, and America. The various aquatic communities (such as benthic and planktic algae, zooplankton and macroinvertebrates) of these ecosystems are exposed to extreme physical and chemical stress (strongly alkaline pans with high conductivity, nutrient concentration, turbidity, and diurnal temperature variation) (Boros, 2013; Stenger‐Kovács et al., 2014), all of which may play a decisive role in selection of a given species (Horváth et al., 2014) able to survive under such circumstances (Pálffy et al., 2014). This strong environmental filter causes a low degree of α‐diversity in alkaline lakes, not only in the case of benthic communities (Stenger‐Kovács, Hajnal, Lengyel, Buczkó, & Padisák, 2016), but also in planktic communities (Nkambo et al., 2015; Vidaković et al., 2019; Vignatti, Paggi, Cabrera, & Echaniz, 2012). However, the degree of β‐diversity found in these communities, primarily determined by the environmental variables, is high due to species turnover (Szabó, Lengyel, Padisák, Vass, & Stenger‐Kovács, 2018); this is true even in sodic anthropogenic, bomb crater ponds (Vad et al., 2017). The conservation of saline lakes is essential if the loss of biodiversity and the disappearance of these unique habitats are to be limited (Williams, 2002).

The main aim of this study was to assess the effects of environmental variables (conductivity, pH, dissolved oxygen, temperature, nutrients [P and N forms], HCO_3_^‐^, CO_3_^2‐^, SO_4_^2‐^, Cl^‐^) and compare these with the individual effect of the geographical (regions) and limnoecological factors (watercolor, substrate, status, hydrological phase, and season) on benthic diatom diversity patterns in soda pans. In order to achieve this, a functional, trait‐based approach has been adopted. In this way, the applicability of functional diversity as an element of ecological status assessment and conservation planning is evaluated, along with the degree to which factors such as adequate sampling time and substrate selection can modify the final results of a status assessment. Two hypotheses were adopted: (a) functional diversity will be an effective indicator of the most characteristic environmental variables, and consequently, of the ecological/conservational status of soda pans, and (b) the individual effects of spatial as well as limnoecological factors on diatom functional diversity will be less pronounced than that of extreme environmental constraints.

## 2 MATERIALS AND METHODS

### 2.1 Sample collection and background variables

**Figure 1:**
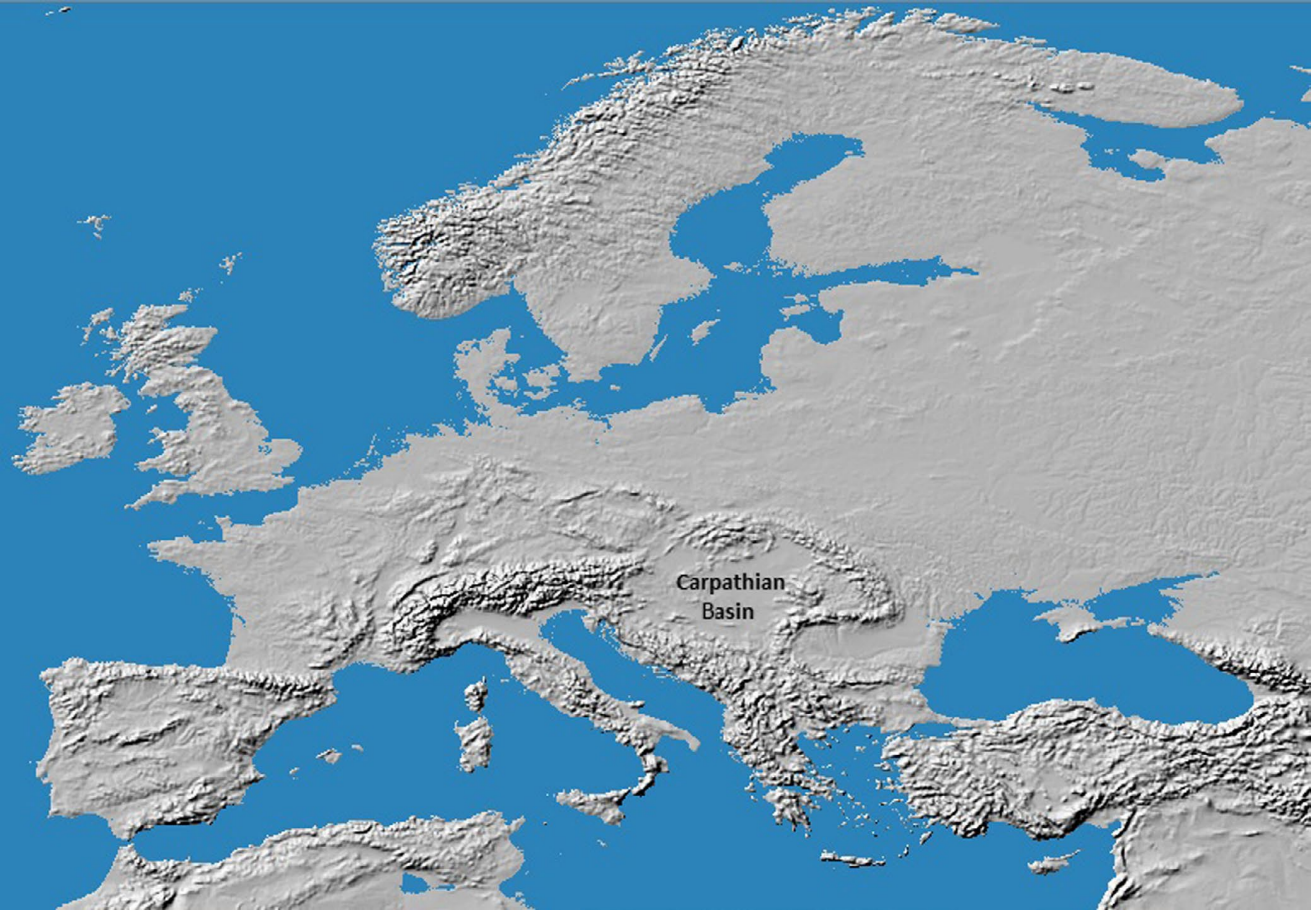
Location of the Carpathian Basin in Europe

A total of 257 diatom and water samples were collected from 32 soda pans (Table 1) over a ten‐year period (2006–2015) in the Carpathian Basin (Central Europe) (Figure 1). Soda pans were categorized by region, status, and watercolor, and the samples by season, hydrological phase, and substrate type. Samples were collected from two main regions of the Carpathian Basin (Central Europe): Fertő‐Hanság and the Danube‐Tisza Interfluve. In contrast to the pans of the Danube‐Tisza region, which may be characterized as having a natural or degraded status (Table 1), the pans of the Fertő‐Hanság region consist of both natural and reconstructed lakes undergoing active conservation activities (Table 1; Stenger‐Kovács et al., 2016). Degraded pans (*n* = 6) were excluded from the analyses of the status effect because of their underrepresentation.

**Table 1:** The status, watercolor, number of the hydrological phases and samples of the studied soda pans from two regions of Central Europe (D, degraded; DT, Danube‐Tisza Interfluve; FH, Fertő‐Hanság region; N, natural; na, no data; RA, reconstructed pans)

Region	Status	Color	Phases	Number of the samples	Name of the pond	Total number of the samples
DT	D	na	na	1	Hattyús‐szék	112
	D	na	na	1	Kisréti‐tó	
	D	na	na	1	Kondor‐tó	
	D	na	na	1	pirtói Nagy‐tó	
	D	na	na	1	Szarvas‐tó	
	D	turbid	4	1	Szappan‐szék	
	D	turbid	4	1	Szívós‐szék	
	N	color	2	13	Bába‐szék	
	N	color	2	21	Sósér	
	N	na	na	1	Ősze‐szék	
	N	turbid	4	12	Böddi‐szék	
	N	turbid	4	13	Bogárzó	
	N	turbid	4	1	Büdös‐szék	
	N	turbid	4	1	pusztaszeri Büdös‐szék	
	N	turbid	4	1	Csárda‐szék	
	N	turbid	4	1	Fehér‐szék	
	N	turbid	4	1	Fülöp‐szék	
	N	turbid	4	1	kardoskúti Fehértó	
	N	turbid	4	21	Kelemen‐szék	
	N	turbid	4	18	Zab‐szék	
FH	N	turbid	na	3	Herrnsee	145
	N	turbid	na	3	Kirchsee	
	N	turbid	na	3	Neubruch	
	N	turbid	na	3	Untersee	
	N	turbid	na	3	Zicklacke	
	N	turbid	na	2	Albersee	
	RA	color	na	3	Cikes	
	RA	transitional	na	34	Borsodi‐dülő	
	RA	transitional	na	5	Pap‐rét	
	RA	turbid	na	54	Legény‐tó	
	RA	turbid	na	32	Nyéki‐szállás	

The sites were classified into three pan turbidity types, this being a prominent feature of sodic lakes, following Boros (2013): (a) colored, (b) turbid, and (c) transitional watercolor (Table 1). A pan is colored if humic materials and is turbid if suspended particles contribute a minimum of 55% to light extinction. The watercolor is transitional if the dissolved humic matter and the suspended particles contribute roughly equally to the light extinction (Boros, 2013).

On the basis of the optical categorization of the pans, various cyclic patterns were determined: filling and concentrated phases for the colored types and filling, diluted, drying and concentrated for the turbid types (Lengyel, Pálmai, Padisák, & Stenger‐Kovács, 2019). The sampling times and their frequency depended on the water supply of these intermittent lakes (Stenger‐Kovács et al., 2016, 2014). The sampling date was matched to the four seasons of the temperate climate. (Detailed maps of and information concerning these lakes may be found in the studies by Stenger‐Kovács et al. (2014, 2018), Stenger‐Kovács et al. (2016) and Lengyel et al. (2016)).

The choice of substrate (mud/macrophyte) and sampling sites followed the recommendations of King, Clarke, Bennion, Kelly, and Yallop (2006). Samples were taken at a water depth of 5–10 cm close to the shorelines of the pans. Diatoms were collected from the macrophytes using toothbrush and were collected from mud by pipetting ~10 cm^3^ of the superficial layer of the pan sediments (Cochero, Romaní, & Gómez, 2013).

### 2.2 Laboratory analyses

Diatom samples were preserved in ethanol and were kept at pH ~7–8 by cc. HCl, thereby avoiding the dissolution of the silica walls. A hot hydrogen peroxide treatment was applied to oxidize the protoplasms (CEN, 2003). Diatom silica valves were embedded in Pleurax^®^ resin. Permanent slides were analyzed under a light microscope (Zeiss Axiovert A1, plan‐apochromat objective with differential interference contrast) and a scanning electron microscope (Hitachi S‐2600N). A minimum of 400 valves were identified at the highest possible taxonomic resolution (Stenger‐Kovács & Lengyel, 2015).

Conductivity, pH, dissolved oxygen, and temperature were measured in the field using an HQD40d Hach Lange multimeter. Other water chemical variables were analyzed in the laboratory with the use of UV/VIS spectrophotometry (SO_4_^2−,^ NO_2_^−^, NH_4_^+^, and TP: total phosphorous) and closed reflux titrimetric (HCO_3_^‐^ and CO_3_^2−^) methods (APHA, 1998; Wetzel & Likens, 2000). Cl^−^ and NO_3_^−^ content were determined using an HQ40d Hach Lange multimeter equipped with ISECI181 and ISENO3181 ion‐specific probes. Dissolved inorganic nitrogen (DIN) was calculated as the sum of NO_3_^−^‐N, NO_2_^−^‐N, and NH_4_^+^‐N.

### 2.3 Statistical analyses

For all sampled material, 35 traits in four trait categories were used in the calculation of the functional diversity indices: (a) diatom ecological guilds (Passy, 2007a; Rimet & Bouchez, 2012); (b) cell size; (c) length/width ratio (e.g., Tapolczai, Bouchez, Stenger‐Kovács, Padisák, & Rimet, 2^2017^); and (d) ecomorphological groups; B.‐Béres, 2016) (Appendix 1). These traits had been tested previously and adopted as applicable indicators of ecosystem functioning in soda pans (Stenger‐Kovács et al., 2018; Appendix 1).

RDA analyses using the forward and backward selection method were used to identify those geographical and limnoecological factors (Appendix 1) and environmental variables which have a significant effect on functional diversity. A further aim here was to study the explained variance of functional diversity metrics by the selected environmental variables as an illustration of the explanatory strength of the trait‐based approach. RDA was applied with the use of a variance matrix and tested using ANOVA, running 999 permutations. The individual effect of the various factors (with region as the geographical factor, and color, substrate, status, hydrological phase, and season as the limnoecological factors) on overall diatom distance‐based functional diversity against the environmental variables was examined in separate variation partition analyses.

Different components of functional diversity were compared with the main driving factors selected via the variation partitioning method using Kruskal–Wallis test with Holm correction, and these were as follows: FRic, functional richness; FDiv, functional divergence; FDis, functional dispersion; RaoQ, Rao's quadratic entropy; FGR, a posteriori functional group richness; and FEve, functional evenness. One Kruskal–Wallis test per predictor was applied, because of the different number of the available data in the case of the main factors. In the statistical analyses, a square root transformation was used for the diatom relative abundance data, while functional diversity indices and the water chemical variables were standardized.

Multivariate linear models (Fox & Weisberg, 2018) were constructed, and their significance levels tested to determine the sensitivity and applicability of the indices. Full models were reduced employing the backward method based on the AIC (Akaike's information criterion) values to select the master variables determining the different functional diversity indices. The similarity of the strengths of the reduced and larger models was checked using the *F* test.

Functional diversity metrics were calculated in the “FD” R package (Laliberté & Legendre, 2010) using the “dbFD” function. Variance partition was performed using the “varpart” function of the “vegan” package (Oksanen et al., 2018).

## 3 RESULTS

**Figure 2:**
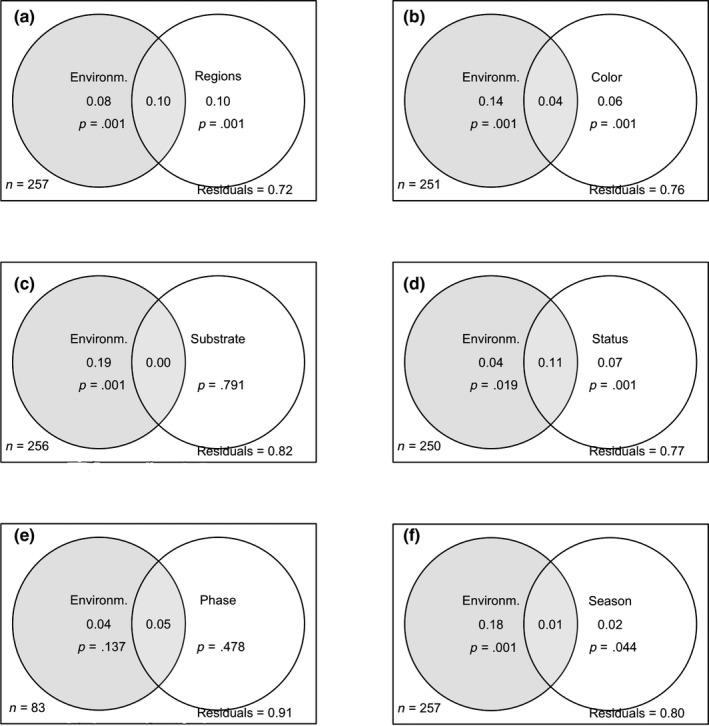
Results of variation partitioning analyses for overall diatom functional diversity based on the environmental variables and (a) spatial factors, (b) watercolor, (c) substrate, (d) status, (e) hydrological phases, and (f) season. Adjusted *R* ^2^ values, significance levels (*p*), and unexplained variances (residuals) are shown in the figure

Using the six functional diversity metrics, variation partitioning showed that environmental variables, region, watercolor, and ecological status had considerable and significant explanatory power with regard to the variations in functional diversity (Figure 2a,b,d). The effects of region and the status were greater than those of the environmental variables (Figure 2a; 0.10; Figure 2d; 0.07). The effect of season alone was less pronounced and very close to the limit of the significance level (Figure 2f; 0.02, *p* = .044). There was hardly any contribution from the effects of the substrate and hydrological phases taken in isolation to the degree of functional diversity (Figure 2c,e).

**Figure 3:**
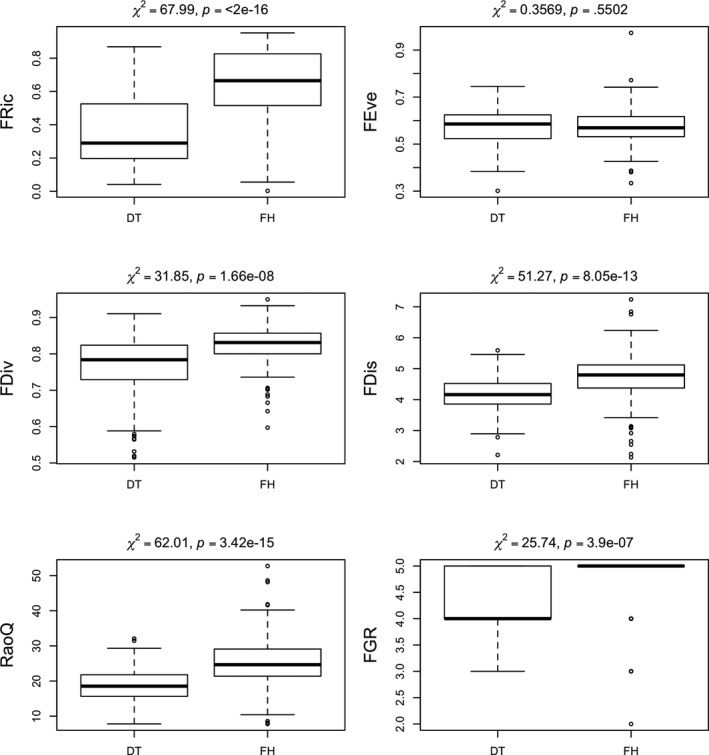
Results of the Kruskal–Wallis test of the six applied functional diversity indices for the different regions (DT‐ Danube‐Tisza Interfluve, FH‐ Fertő‐Hanság region)

Examining the various components of functional diversity on the basis of the factors with significant explanatory value, the response of the indices was different in different regions: On the basis of the Kruskal–Wallis test, in the Danube‐Tisza Interfluve, the index values were significantly lower than those in the Fertő‐Hanság region. Only one index, functional evenness, was not sensitive to variation by region (Figure 3).

**Figure 4:**
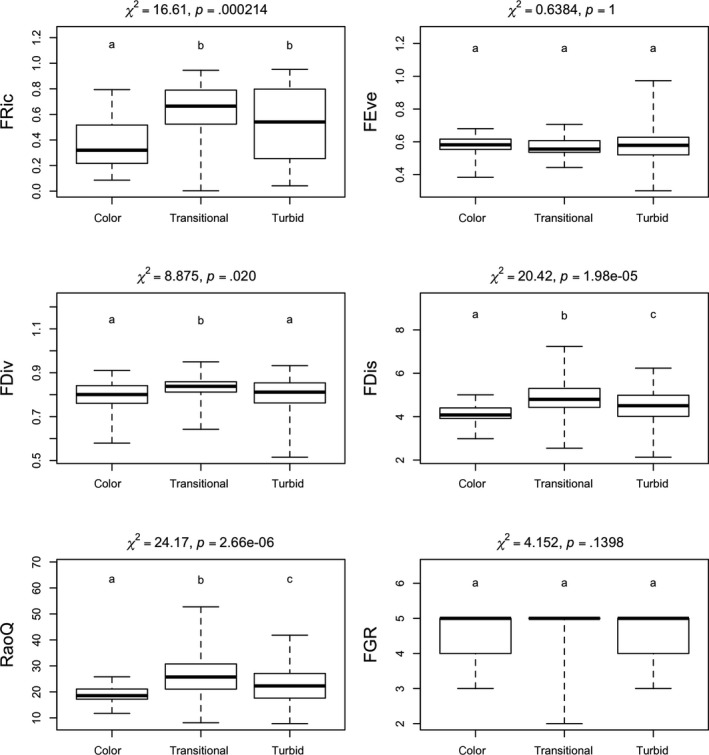
Results of the Kruskal–Wallis test of the six applied functional diversity indices for different watercolor types (groups with the same letters are not distinct, whereas groups with different letters differ significantly)

In the case of the watercolor, the responses of the indices were more varied (Figure 4). The values of RaoQ and FDis differed significantly depending on watercolor. RaoQ and FDis had the lowest values in colored soda pans, while in the transitional pans, they had the highest. FDiv was significantly lower both in the colored and turbid pans. FRic was lower in colored waters and showed no significant variation in value between the transitional and turbid ones. In the values of FEve and FGR, no significant differences were observed (Figure 4).

**Figure 5:**
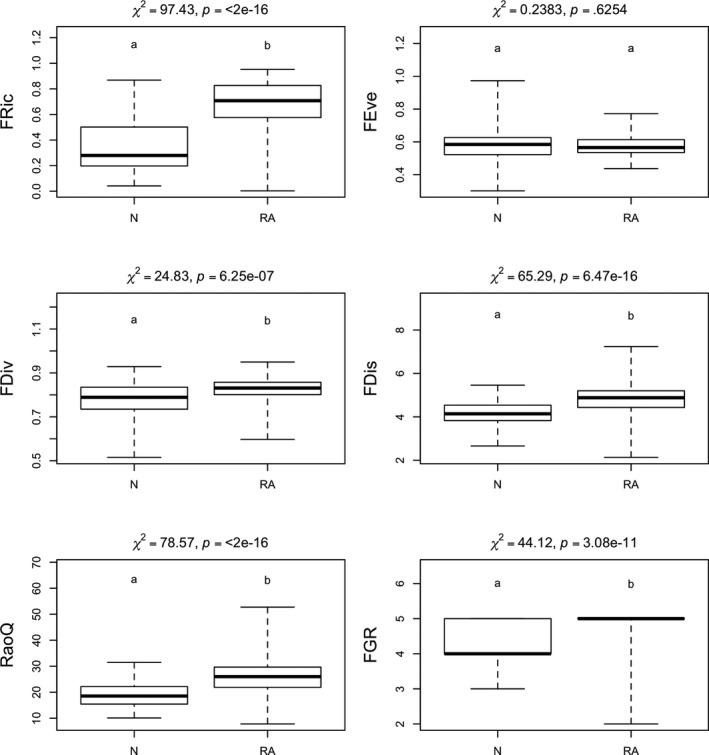
Results of the Kruskal–Wallis test of the six applied functional diversity indices for soda pans with different ecological status (D: degraded, N: natural, RA: reconstructed pans; groups with the same letters are not distinct, whereas groups with different letters differ significantly)

Five indices (FDiv, FDis, RaoQ, FGR, and FDis) differed significantly between natural and reconstructed areas, with lower diversity values indicating the natural status of the soda pans (Figure 5). The FEve values were similar in soda pans with different statuses (Figure 5).

**Figure 6:**
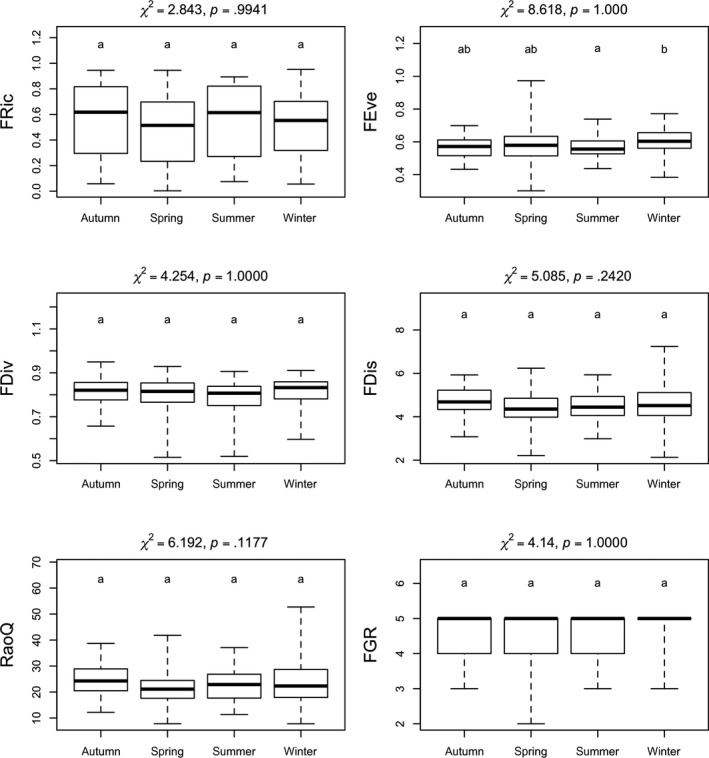
Results of the Kruskal–Wallis test of the six applied functional diversity indices for soda pans in different seasons (groups with the same letters are not distinct, whereas groups with different letters differ significantly)

The individual indices were not sensitive to the seasons, except for FEve, which was significantly different in summer and winter (Figure 6).

**Figure 7:**
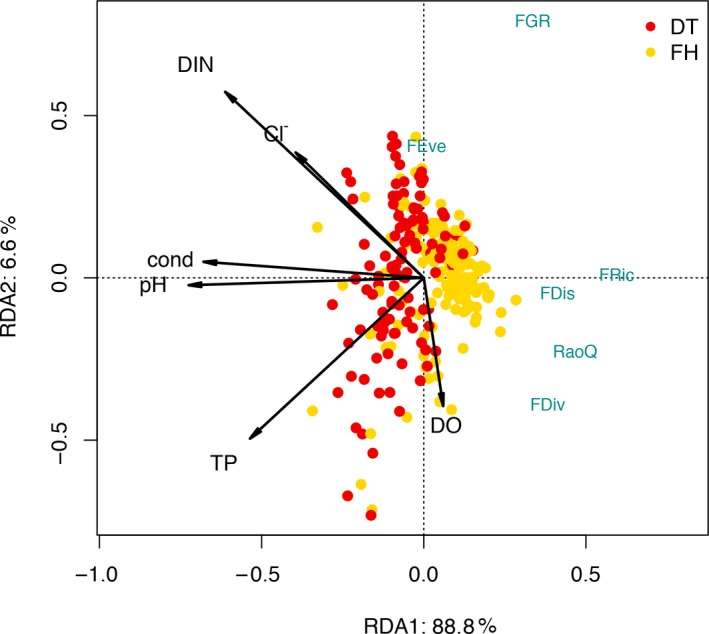
Redundancy analyses (RDA) of the six applied functional diversity indices and the environmental variables (bicarb, HCO_3_^−^; carb, CO_3_^2−^; cond, conductivity; DIN, dissolved inorganic nitrogen; DO, dissolved oxygen; temp, temperature; TP, total phosphorus

Significant effects of the environmental variables on the functional diversity indices were found in the course of the RDA analysis (Figure 7). On the first axis, 89% of the total constrained variance of the functional diversity indices was explained by the environmental variables. DIN, temperature, pH, conductivity, and DO were the main constraints that determined functional diversity to a great extent. After the reduction of the full models containing ten environmental variables, FRic was determined by eight, FDiv, RaoQ, and FGR by seven, and FDis was determined by six variables (Table 2). In the reduced models, the effect of conductivity, pH, HCO_3_^−^, Cl^−^, DIN, and SO_4_^2−^ was significant in the case of FRic; conductivity, pH, DO, and HCO_3_^−^ in FDiv; conductivity, pH, Cl^−^, and DIN in FDis; and conductivity, pH, and DIN in RaoQ. Conductivity, pH, TP, Cl, and SO_4_^2−^ had a significant relationship with FGR.

**Table 2:** Results of the reduced multivariate linear models

	Functional diversity indices	temp	cond	pH	DO	TP	CO_3_^2−^	HCO_3_^−^	Cl^−^	DIN	SO_4_^2‐^	*F*	*p*	*df*
Reduced model	Fric		***	*				**		***	*	17.17	<0.001	250
	FDiv		*	***	***			*				7.607	<0.001	251
	FDis		***	**					***	**		9.014	<0.001	252
	RaoQ		***	*						***		9.934	<0.001	251
	FGR		**	**		**			**		*	7.409	<0.001	251

Note

Gray background: environmental variables that the models contained.

Abbreviations: temp, temperature; cond, conductivity; DO, dissolved oxygen; TP, total phosphorus; DIN, dissolved inorganic nitrogen.

* *p* < .05;

** *p* < 0. 01;

*** *p* < .001.

## 4 DISCUSSION

Soda pans are characterized by low functional diatom diversity similar to low species diversity (diatom α‐diversity) (Stenger‐Kovács et al., 2016;) and low phylogenetic diversity of zooplankton communities (Horváth et al., 2014). The main reasons for the low functional diversity are (a) a low degree of habitat heterogeneity (Stark, Lehman, Crawford, Enquist, & Blonder, 2017) as a strong filter (Anacker & Harrison, 2012) and (b) the harsh environment (Heino, 2005). In other words, these extreme ecosystems impose highly stressful conditions on the biota, making this a selection force for species, and consequently a strong driver of the selection of suitable traits (Abonyi et al., 2018; Teittinen, Weckström, & Soininen, 2018). Only functionally similar species can survive, causing low functional diversity not only in these saline and extreme ecosystems, but also in intermittent streams, where droughts (extreme events) also have negative effects on the functional diatom diversity (B.‐Béres et al., 2019). As in terrestrial plant communities, low functional diversity is also characteristic in mountains with specific environmental conditions (Schneider et al., 2017).

Of the factors examined, region, status, and watercolor type were found to have a considerable effect on the functional diversity of soda pans. The effect of region and status was also highlighted with regard to species composition (Stenger‐Kovács et al., 2014) and species‐based diversity metrics (species richness, Shannon diversity, and taxonomic distinctness; Stenger‐Kovács et al., 2016). However, from amongs these factors, region as a spatial effect had the highest degree of success in explaining variance observed in changes in diatom functional diversity, lending further support to the notion that spatial processes have a strong effect on community structure and function (Heino et al., 2015). Consequently, functional trait proportions change spatially not only in the case of other aquatic organisms (e.g., macroinvertebrates, Schmera, Erős, & Heino, 2013), but also in the case of diatoms. This spatial effect on diatom functional diversity as examined from the perspective of several traits exceeded that of the environmental variables on a regional scale. This stands in contrast to what was found on global scale on the basis of the composition of diatom ecological guilds alone (Soininen, Jamoneau, Rosebery, & Passy, 2016) as functional traits. The response of the applied functional diversity indices (with the exception of FEve) was unanimous for the regions: Their values were significantly lower in the Danube‐Tisza Interfluve.

The variance in functional diversity metrics explained by environmental variables was five times higher when a trait‐based approach was applied (95.4%), as against species‐based community analyses (18.1%, Stenger‐Kovács et al., 2014). The strong relationship of functional diversity to environmental variables had previously been highlighted by other studies which drew attention to their important role in shaping functional structure (Li et al., 2019; Teittinen et al., 2018). This means that in soda pans environment selects, consequently, deterministic processes are characteristic (Szabó et al., 2018), and environmental filtering causes a functional convergence pattern; species with similar ecological strategies and adaptations (suited traits) coexist (Cornwell & Ackerly, 2009; Petchey, Evans, Fishburn, & Gaston, 2007; Weiher & Keddy, 1995) as is also the case among periphytic algae in a floodplain conservation area (Bichoff, Osório, Ruwer, Dunck, & Rodrigues, 2018).

The status of soda pans is dependent upon the maintenance of their natural hydrological cycle (Stenger‐Kovács et al., 2016). Drying out is a natural and required feature of the pans (Gavrilović et al., 2018), and this, in turn, can radically reduce functional diversity (B.‐Béres et al., 2019). Under pristine ecological status, not only small species numbers and species‐based diversity (Stenger‐Kovács et al., 2016), but also restricted trait variations can be found in the present study: Motile, small, elongated diatom species are characteristic (e.g., *Nitzschia austriaca* Hustedt, *Nitzschia aurariae* Cholnoky*, Craticula elkab* (O. Muller ex O. Muller) Lange‐Bertalot, Kusber & Cocquyt), indicating the harsh (high conductivity and turbidity, temporary drying out) environment (Stenger‐Kovács et al., 2018). The motility allows the species to change their position to find the “best place” under these unfavorable conditions. The small cell size and this elongated shape further facilitate their movement among the inorganic sediment particles and their ability to hide in the mud. Small size has also been highlighted in planktic communities (Alfonso, Zunino, & Piccolo, 2017; Somogyi et al., 2014) as well as the motile feature (Földi et al., 2018) in other saline lake ecosystems, where species reduce their cell and pore size due to the osmotic stress (Leterme et al., 2010).

Functional diversity metrics displayed significantly lower values in natural soda pans, indicating their pristine features. The diversity values of the degraded pans did not differ either from the natural ones or from those of reconstructed pans, which had significantly higher functional diversity than natural lakes. Disturbed hydrological cycles (e.g., by water abstraction or resupply) can modify limnological variables (e.g., lower conductivity and pH) (Lengyel et al., 2016), potentially leading to less extreme features characteristic of fresh water, and therefore resulting in higher diversity. This result calls attention to anthropogenic activities (Alfonso et al., 2017), including even those undertaken for conservation purposes, which have considerable impacts on biodiversity both on the taxonomic (Heino, 2005; Stenger‐Kovács et al., 2016) and at the functional levels.

As forest shading of streams reduces functional diversity (Taniwaki et al., 2019), the light climate of soda pans on the basis of their color type (Lengyel et al., 2019) had a considerable effect on the trait composition and functional diversity, as also experienced in the diatom community composition of artificial bomb crater ponds (Földi et al., 2018). While the turbidity of lakes reduces light intensity, high levels of humic materials can modify the spectral composition of the incoming light (Kirk, 1994; V.‐Balogh, Németh, & Vörös, 2009). In contrast to other aquatic ecosystems where the light intensity is high and different growth forms can coexist (Passy & Larson, 2011), here only those species with adequate traits can survive, and this results in a low degree of functional diversity. One possible adaptation strategy, besides the chromatic adaptation of algae, might be size as a key trait, since the surface area of small cells is relatively large in proportion to their volume/size, an advantage in the competition for light (Somogyi & Vörös, 2004). Elongated forms can serve as light traps (Stenger‐Kovács et al., 2018) under the low light intensity of soda pans. FDis and RaoQ were the most sensitive indices of watercolor type, since these differed most in the three color types. The low value of the indices in the turbid and colored pans indicated the higher stress caused by high levels of inorganic particles or humic materials, as compared with the transitional ones, in which the amount of these materials was relatively smaller.

In these ecosystems, seasons had a less pronounced effect, and the related hydrological cycle, as well as the substrate type, had no significant effect on functional diversity, in contrast to the case of freshwater, where high water periods support the appearance of a number of periphytic algal species with different traits, thus resulting in a high degree of functional diversity (Dunck, Algarte, Cianciaruso, & Rodrigues, 2016; Dunck, Rodrigues, & Bicudo, 2015). At the taxonomic level, seasonal effects (Lengyel et al., 2016) can also be detected in the benthic diatom, as well as in the planktic communities of saline lakes (Alfonso et al., 2017). However, microhabitat preference (such as substrate type) is negligible at taxonomic levels as a consequence of the extreme environmental conditions (Cejudo‐Figueiras, Álvarez‐Blanco, Bécares, & Blanco, 2011; Lengyel et al., 2016). This result further emphasizes the primarily role of local factors (Bichoff et al., 2018) and of the strong environmental filters on the structure and function of the communities, (Ding et al., 2017; Soininen, 2012) even in saline ecosystems (Horváth et al., 2014).

Of the environmental variables, DIN, temperature, pH, conductivity, and DO were the main determinants of the functional diversity metrics as revealed by the RDA analyses. This stands in contrast to the taxonomic diatom assemblages that were chiefly determined by conductivity, bicarbonate, and sulfate concentration (Stenger‐Kovács et al., 2014) in natural ponds, while, salinity, pH, and turbidity dominated in artificial saline ones (Földi et al., 2018). All specific functional diversity indices were sensitive to the most important environmental variables of soda pans—conductivity and pH—as has also been found in subarctic ponds (Teittinen et al., 2018). Furthermore, most (FRic, FDis, and FGR) were type‐specific, that is, the response of the indices was also based on the dominant, basic anions (chloride and sulfate) in the pans. Comparing these to the species‐based diversity metrics (species richness, Shannon diversity, and taxonomic distinctiveness) (Stenger‐Kovács et al., 2016), the functional diversity indices were more complex indicators since they integrated the effects of more environmental variables (from three to five, instead of two or three), and this plays a crucial role in the indication of environmental changes. On the basis of the strong correlation between these key variables, they are very effective and informative metrics (for macroinvertebrates, see He et al., 2015). Of the functional diversity metrics studied, FGR, FRic, FDis, and FDiv proved to be the most useful for assessing the ecological status and conservation value of soda pans. FEve was not related to changes in the environment, as has been shown in the case of diatoms in tropical headwater streams (Taniwaki et al., 2019), and over the long term by phytoplankton communities in a large river (Abonyi et al., 2018).

## 5 CONCLUSIONS

As in all terrestrial ecosystems (Díaz & Cabido, 2001), functional diversity can be shown to be a proper tool to aid the understanding of patterns and processes along the environmental and spatial gradients of aquatic ecosystems such as soda pans. This trait‐based method was effective in indicating environmental changes and degradation processes; the variance of the functional diversity metrics explained by the environmental variables was five times higher than the taxonomical one. Furthermore, functional diversity metrics were type‐specific and independent of substrates and seasonal influences. This may well have major importance in ecological status assessments and conservation planning. Diatom trait‐based functional diversity indices proved to be both more complex and more applicable indicators as compared with traditional taxonomical diversity metrics because they integrate the effects of a greater number of master variables of these unique environments. Consequently, the approach applied here makes conservation of this habitat type possible in a functional way and potentially globally.

## CONFLICT OF INTEREST

The authors have no conflict of interests to declare.

## AUTHORS’ CONTRIBUTIONS

CS‐K formulated the idea, selected the methods, and wrote the first draft of the manuscript. CS‐K and EL collected the samples and identified the diatom species with a light microscope with the help of KB. JK and CS‐K were responsible for the statistical analyses. JP provided detailed knowledge about functional classifications. All authors contributed substantially to revisions.

## Data Availability

Data for diatom abundances and background variables are deposited in the Mendeley Digital Repository: http://dx.doi.org/10.17632/bv8p7nfb27.1

## APPENDIX 1

**Table:**

Traits	Function	Soda pans	Toward freshwater	Typical species examples
Biovolume (S)				
S1 (<100 µm^3^)	Cell size influence on their distribution^1^ Physical disturbances (smaller species with greater resilience)^2^ Increasing salinity (reduction of cell size and pore size)^3^ Light availability (large species under higher light intensity)^4^ Easier movement among inorganic particles of small species^5^	X		*Nitzschia austriaca* (motile, MS1)
S2 (100–300 µm^3^)				
S3 (300–600 µm^3^)				
S4 (600–1500 µm^3^)			X	*Ctenophora pulchella*
S5 (>1,500 µm3)				
Length/width ratio (L/W)				
LW1 < 2	Elongated taxa with small L/W in polluted habitats with high shear stress^6^ High conductivity (LW2, LW3)^5^ Easier hiding or moving among mud particles of more elongated taxa^5^ Elongated taxa are light traps in light‐limited area^5^			
LW2 (2–4)		X		*Anomoeoneis sphaerophora* (motile, MLW2)
LW3 (4–6)		X		*Nitzschia salinarum* (motile, MLW3)
LW4 (6–12)				
LW5 (12–20)				
LW6 ( >20)				
Ecological guilds				
High profile	Lower conductivity^5^ High light intensity^7^			*Bacillaria paxilifera*
Low profile	Lower conductivity^5^ Frequent disturbance events^10^ Low nutrient content^10^		X	*Amphora copulata*
Motile	Resource rich habitats^4^ High turbidity^6^ High salinity^5,9^ Siltation and land use^8^ Motility in fine sediment particles^8^ Water abstraction^4^ Drying phases^9^	X		*Nitzschia bergii* *Nitzschia supralitorea*
Planktic	Lower conductivity^5^		X	*Aulacoseira ambigua*
Ecomorphological groups				
MS1‐MS5; HS1‐ HS5; LS1‐LS5; PS1‐PS5	Size ‐dependent guilds separation make it possible to detect more pronounced relationship between traits and environmental variables^11^			

Applied traits and their functions according to the relevant papers (^1^Heino & Soininen, 2006; ^2^Passy, 2007b; ^3^Leterme et al., 2010; ^4^Lange et al., 2011; ^5^Stenger‐Kovács et al., 2018; ^6^Taploczai et al., 2017; ^7^Trábert et al., 2017; ^8^Smucker and Vis, 2010; ^9^Kókai et al., 2015; ^10^Novais et al., 2014; ^11^B.‐Béres et al., 2016). Characteristic traits with example species in pristine soda pans and towards freshwaters features signed by X.

Transmission electron microscopic (TEM) photo about the characteristic motile, small, elongated diatom species from a natural soda pan (Zab‐szék, Hungary).

Results of the RDA analyses of the functional diversity and geographical and limnoecological factors after backward and forward selection.

**Table:**

Factors	Sample number	AIC	*F*	*p*
Region	251	1941	25.44	.005
Status	251	1931.5	6.86	.005
Season	251	1928.5	2.96	.005
Colour	251	1925.8	3.28	.005
Substrate	251	1923.2	3.17	.005
Phase	83	642.6	2.84	.005
